# Brain mechanics and cognition: the role of rodent MR elastography in identifying cellular mechanisms

**DOI:** 10.3389/fcogn.2026.1943713

**Published:** 2026-09-30

**Authors:** Katrina A. Milbocker, Sabrina S. Vander Wiele, Curtis L. Johnson

**Affiliations:** Department of Biomedical Engineering, University of Delaware, Newark, DE, United States

**Keywords:** magnetic resonance elastography, memory, preclinical neuroscience, stiffness, viscosity

## Abstract

Magnetic resonance elastography (MRE) non-invasively quantifies brain tissue mechanical properties and has revealed an intriguing relationship between regional viscoelasticity and cognitive function across the lifespan. However, the biological basis of this relationship remains poorly understood as it is not yet clear which microstructural features generate the mechanical signal, whether brain mechanics are primarily structural or also dynamic on cognitive timescales, or how generalizable the current relationships observed in humans are across different brain regions and functions. We argue that resolving these questions requires preclinical MRE in rodents, where mechanical measurements can be linked directly to complementary analyses of cellular components and where structure and function can be experimentally perturbed. Although rodent MRE is well-established and has been productively applied to disease models, it has been little used in the contexts in which the human MRE-cognition findings are most concentrated including normal development and aging and cognitive variation in the otherwise-healthy brain. We review the human MRE-cognition literature, summarize what preclinical MRE has revealed to date, and outline experimental frameworks organized around the open questions through which rodent MRE could begin to clarify why brain mechanics relate to brain function.

## 1 Introduction

Magnetic resonance elastography (MRE) non-invasively quantifies the mechanical properties of living tissue by imaging the propagation of shear waves, yielding *in vivo* measures of viscoelasticity that reflect the microstructural organization of the underlying tissue (Muthupillai et al., 1995; Manduca et al., 2021; Sack, 2023). Applied to the brain, MRE has been used in several clinical populations, notably in Alzheimer's disease and related dementias where the brain is softer with neurodegeneration (Murphy et al., 2019; Arani et al., 2021; Hiscox et al., 2021). In addition to its use in understanding neuropathology, MRE has shown sensitivity to aging and development (Sack et al., 2011; Arani et al., 2015; Hiscox et al., 2018; Johnson and Telzer, 2018; McIlvain et al., 2022). Year-over-year decline in brain stiffness is one of the most robust findings in the brain MRE literature (Hiscox et al., 2021) and recent machine learning-based brain age models incorporating MRE data have shown improved performance (Claros-Olivares et al., 2025; Träuble et al., 2025). Interestingly, brain MRE studies have uncovered relationships between regional mechanical properties—i.e., tissue stiffness and damping ratio—and performance on tests of brain function and cognition, such as hippocampal-based memory (Schwarb et al., 2017, 2019, 2016), often with greater sensitivity than volumetric or diffusion measures.

Which cells and matrix constituents give rise to the mechanical properties MRE measures, however, remains poorly understood. MRE provides metrics of tissue mechanics that are calculated from imaged shear wave displacements using an inversion algorithm, and are often reported as shear stiffness and damping ratio (Manduca et al., 2021). Stiffness refers to the resistance of the tissue to deformation while damping ratio reflects relative viscosity or how much mechanical energy is absorbed by the tissue (McIlvain, 2024). In the brain, these mechanical properties are known to emerge from the combined biomechanical characteristics of neurons, glia, myelin, vasculature, and the extracellular matrix, and their organization in brain tissue (Bergs et al., 2024). But these features have not been explored in the context of brain mechanics and brain function jointly, and which of them link the two is unknown. Making sense of these mechanical structure-function relationships is further compounded by the unknown specificity and generalizability of the relationship, and how it evolves across multiple timescales.

Resolving these questions requires a measurement system in which MRE outcomes can be linked directly to the cellular and molecular features that produce them. Preclinical MRE in rodents pairs non-invasive *in vivo* imaging with direct histological, molecular, and genetic interrogation of the same tissue. Rodent brain MRE is now a well-established method (Bayly and Garbow, 2018; Bigot et al., 2018), and has been used productively to characterize mechanical signatures of significant neurological conditions through disease models (Schregel et al., 2012; Freimann et al., 2013; Hain et al., 2016; Majumdar and Klatt, 2021; Silva et al., 2024). Indeed, much of what we know about the microstructural basis of brain mechanics comes from changes in disease models that produce demyelination and inflammation (Riek et al., 2012; Schregel et al., 2012; Millward et al., 2015; Silva et al., 2021). Far less attention has been paid to the rodent brain in the contexts in which the human MRE-cognition relationships are most often observed including normal development and aging, and no studies to date have also performed behavioral testing to understand neural function in the otherwise-healthy brain. Further, rodent models offer the opportunity for experimentally-controlled manipulations to modify both brain mechanics and brain function to mechanistically interrogate their association.

In this Perspective, we review what is known and unknown about the link between brain tissue mechanics and cognition, and describe how rodent brain MRE studies can be positioned to fill key gaps in knowledge about this relationship.

## 2 Brain tissue mechanics as a novel correlate of cognitive performance in humans

The first and most studied relationship observed between brain mechanics measured with MRE and cognitive function is between hippocampal mechanical properties and memory. Schwarb et al. reported that in young adults, hippocampal damping ratio—a measure of the ratio of relative viscous-to-elastic behavior (McIlvain, 2024)—was strongly associated with relational memory performance on a spatial reconstruction task, whereas hippocampal volume was not, suggesting that MRE captures unique microstructural information relevant to memory function (Schwarb et al., 2016). This relationship was replicated in a larger study (Schwarb et al., 2017) and similar findings were observed with other hippocampal-dependent cognitive tasks (Schwarb et al., 2019; Hiscox et al., 2020b). By harnessing high-resolution methods to examine individual hippocampal subfields (Delgorio et al., 2021), Delgorio et al. found that subfield stiffness—rather than damping ratio—predicted memory performance in a sample of adults ages 23–81 years, with stiffness of subfields differentially correlating with performance on a range of memory tasks, ostensibly owing to the regional specificity of the different hippocampal subfields to different aspects of human memory (Delgorio et al., 2022).

Beyond the hippocampus, brain MRE studies reveal regionally specific structure-function relationships. Johnson et al. documented that damping ratio of the orbitofrontal cortex was significantly associated with performance on a figures series task of fluid intelligence (Johnson et al., 2018). Notably, in this same sample, hippocampal damping ratio was associated with relational memory performance, as before, but there was a double dissociation between structures and functions. Similarly, Schwarb et al. found separate associations between hippocampal damping ratio and relational memory and ventromedial prefrontal cortex damping ratio and novel rule learning with both measures taken from a single task (Schwarb et al., 2019). This regional specificity extends to other populations and functional domains, including sequence learning in older adults (Heselton et al., 2025), vocabulary in children (Schneider et al., 2022), and behaviors such as risk-taking tendencies in adolescents (McIlvain et al., 2020).

Identifying these structure-function relationships remains an evolving effort across brain regions and cognitive domains (Figure 1), and interestingly which measure carries the association—stiffness or damping ratio—can shift with region, task, and population age range. Importantly, stiffness and damping ratio capture fundamentally different aspects of tissue mechanical behavior and are therefore likely sensitive to distinct microstructural features. Stiffness is thought to be primarily governed by the density and organization of load-bearing structural elements, including number of neurons, glial cell density, and extracellular matrix composition (Sack et al., 2013). The damping ratio, by contrast, reflects the balance between viscous and elastic behavior of a viscoelastic material, and is often thought to reflect the organization and complexity of brain microstructure (Sack et al., 2013; McIlvain, 2024). Because these parameters have partially distinct microstructural underpinnings, they may relate to cognitive function through different pathways and may not change in parallel across brain regions or conditions. For instance, a process that primarily disrupts oligodendrocytes and myelination may preferentially affect damping ratio, while one characterized by edema or neuroinflammation may more prominently alter the stiffness. This distinction is critical for interpreting associations between tissue mechanics and cognition, as the parameter most sensitive to a given biological process may vary by region, population, and disease stage. Collectively, this line of work is directed at determining how MRE can sensitively assess tissue structure and predict meaningful outcomes in health and disease, or response to intervention.

**Figure 1:**
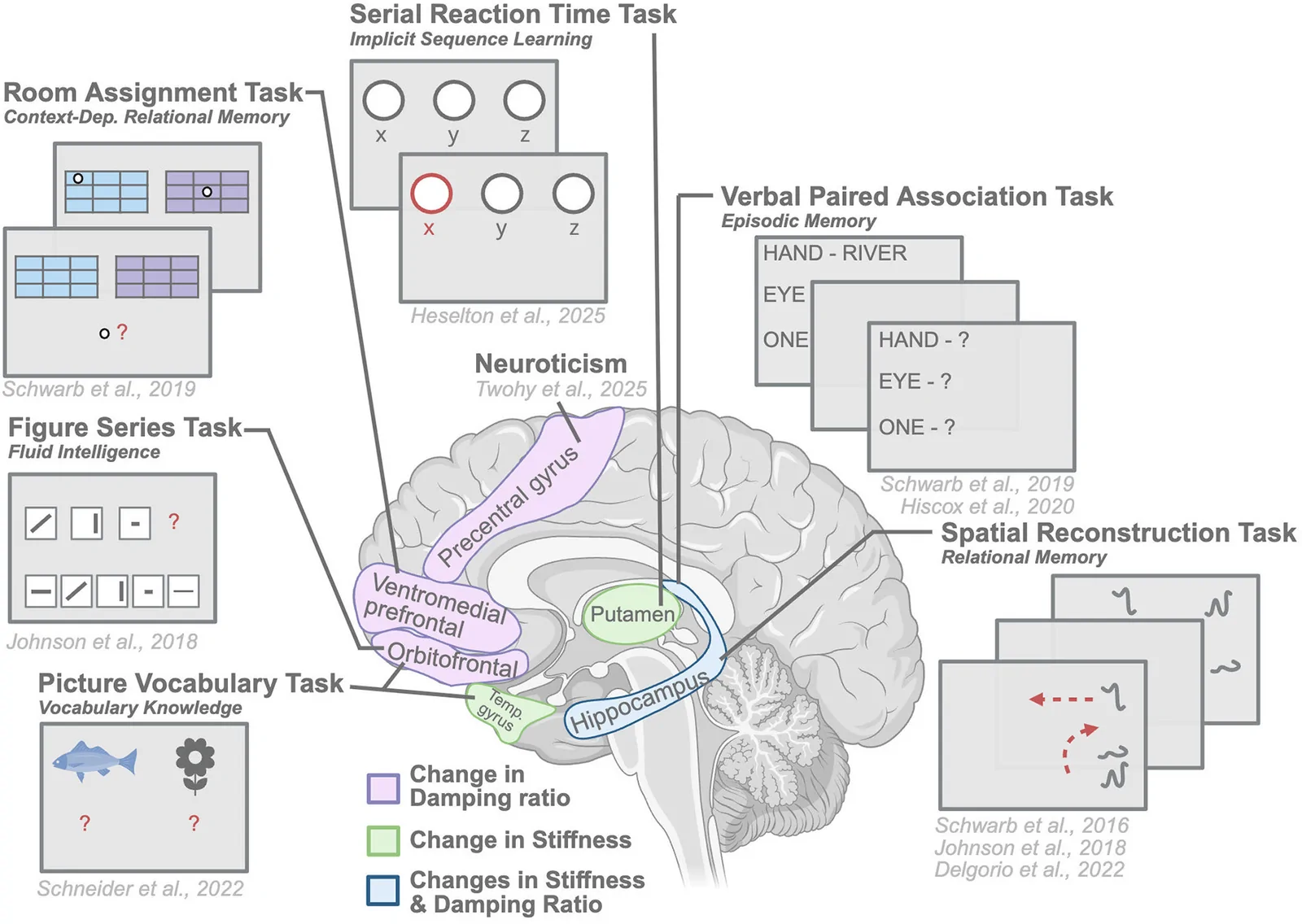
Summary of behavioral tasks used to probe cognitive capacity in human brain MRE studies and 631 their regional correlations with brain mechanical properties. Cognitive performance correlates with regional 632 tissue damping ratio (purple), stiffness (green), or both (blue), depending on the study. “Regional brain tissue mechanics associated with MRE outcomes” by K.A. Milbocker created in BioRender (https://shorturl.at/xmD1i) is licensed under CC BY 4.0.

## 3 Aging and lifestyle factors as modifiers of the mechanical structure-function relationship

Much of the research into mechanical structure-function relationships has had an overarching goal of establishing MRE as a predictor of cognitive brain health, often with an ultimate application in aging and neurodegenerative conditions. Aging is characterized by major changes in tissue biology including widespread cell death, elevated inflammation, regional atrophy and cortical thinning, arteriosclerosis, reduced blood and glymphatic flow (Benveniste et al., 2019), and decomposition of the tissue matrix (Yankner et al., 2008). Age-related tissue degeneration is captured as widespread brain softening with MRE where global brain stiffness declines between 0.3%−1.0% per year, depending on neuroanatomical region, while damping ratio also increases (Hiscox et al., 2021). While the original structure-function MRE studies were on young adults (Schwarb et al., 2016, 2017, 2019; Johnson et al., 2018), one study examined the hippocampal damping ratio and memory function relationship in a population of older adults (Hiscox et al., 2020b) and found a consistent relationship. However, in a study on hippocampal subfields with a population spanning adulthood, there was no association with damping ratio but rather with stiffness (Delgorio et al., 2022). In a study of middle-aged and older adults, Heselton et al. (2025) found that sequence learning was associated with stiffness but not damping ratio. These results suggest that the mechanical structure-function relationship may be modulated by age itself, complicating the use in predicting age-related cognitive decline. Nevertheless, Pavuluri et al. demonstrated that medial temporal lobe stiffness indeed predicts subsequent cognitive decline in aging independently of amyloid burden and cortical thinning (Pavuluri et al., 2025). An additional study of note found that damping ratio of specific cortical regions was correlated with neuroticism in a population of older adults (Twohy et al., 2025). This is a notable extension of similar relationships to personality traits, and that neuroticism is a risk factor for the development of Alzheimer's disease (Segerstrom, 2020), and MRE may have further predictive value in this context.

Emerging evidence suggests that brain tissue mechanical properties are not fixed features but are actively modulated by lifestyle factors, with direct implications for cognition in adulthood and in aging. Aerobic fitness, a well-established modulator of neurogenesis, angiogenesis, and myelination that protect cognitive capacity has been examined in this context previously. In young adults, higher lifetime aerobic fitness is associated with more favorable hippocampal viscoelasticity (lower damping ratio), and hippocampal damping ratio statistically mediates the relationship between fitness and relational memory (Schwarb et al., 2017). In another study, a 12-week exercise program increased hippocampal stiffness in adults with multiple sclerosis alongside improvements in memory function (Sandroff et al., 2017). These findings point to a straightforward model in which fitness preserves or enhances tissue stiffness to support cognition. Declining cardiometabolic health, a similar factor known to affect brain health, is also correlated with brain tissue mechanical changes. Circulating HDL cholesterol was found to be positively correlated with hippocampal stiffness (Sanjana et al., 2021), while in another study higher arterial stiffness and cerebrovascular pulsatility index were negatively associated with hippocampal stiffness (Sanjana et al., 2023). Pavuluri et al. found that brain stiffness mediated the relationship between vascular disease measures and cognitive function in aging adults (Pavuluri et al., 2024).

While vascular health and function clearly contribute to alterations in tissue stiffness, whether this represents the dominant mechanism remains uncertain. These associations are likely further modulated by timescale from short term impacts of changes in blood volume to longer term remodeling of brain tissue from changes in blood flow. Mechanistic studies in rodent models could help disentangle these contributions and clarify which metabolic changes most strongly drive alterations in tissue stiffness, informing future clinical applications of brain MRE.

## 4 Basic findings from the emerging field of rodent brain MRE

Identifying the cellular and microstructural basis of brain mechanics cannot be resolved with *in vivo* human studies. While there are several studies mechanically examining human brain tissue *ex vivo*, these are limited by the small samples and heterogeneous donors (Chatelin et al., 2012; Forte et al., 2017), and confounded by sensitivity to testing conditions and the rapid change in brain mechanics following death (Weickenmeier et al., 2018; Wang et al., 2024; Mojumder et al., 2026). Instead, preclinical brain MRE studies on rodents have served as a primary tool for this purpose, as *in vivo* MRE measures can be paired directly with *ex vivo* tissue analysis.

Neurodegenerative disease models are the most widely studied using rodent brain MRE, with studies often employing a pharmacologically-induced pathology or genetically modified animal model. Across degenerative disease models, including Alzheimer's (Murphy et al., 2012; Majumdar and Klatt, 2021) and Parkinson's disease (Hain et al., 2016), MRE reveals regional softening generally consistent with findings in the human brain (Lipp et al., 2018, 2013; Murphy et al., 2016; Gerischer et al., 2018; Hiscox et al., 2020a). Such softening is associated with amyloid deposition, dopaminergic neurodegeneration, and tissue remodeling (Palotai et al., 2022). In a study using a model of multiple sclerosis with cuprizone-induced demyelination, MRE captured reduced brain stiffness and damping ratio associated with myelin loss and oligodendrocyte death (Schregel et al., 2012). Another multiple sclerosis model via induction of experimental autoimmune encephalomyelitis (EAE) to model neuroimmune dysfunction with resulting demyelination results in viscoelastic changes that correspond with inflammatory cell infiltration (Riek et al., 2012; Millward et al., 2015; Silva et al., 2021). Lesion-based studies further reveal localized matrix remodeling, particularly disruption of perineuronal nets (Wang et al., 2020b; Silva et al., 2024). In a model of Fetal Alcohol Spectrum Disorders (FASD) achieved in rats via postnatal alcohol exposure, we also found reduced brain stiffness consistent with disrupted perineuronal nets in adolescence that resolved into early adulthood (Milbocker et al., 2024b; Williams et al., 2026).

While many of these works with preclinical disease models are foundational in providing a basis for interpreting brain mechanics, the MRE literature studying otherwise healthy rodents is comparatively thin—this is also context in which the human MRE-cognition findings are concentrated. In a seminal paper by Guo et al., MRE was performed longitudinally on young mice finding regional patterns of brain tissue changes in stiffness and damping ratio, which were correlated with a variety of proteins determined via mass spectrometry, supporting multiple, interrelated contributions to changing mechanics in the developing brain (Guo et al., 2019). Such multifaceted findings exemplify the complexity of how tissue microstructural composition and organization combine to result in mesoscale tissue mechanics, and how precise experimental designs are necessary to pinpoint the cellular source of observed imaging effects.

There is also a need to study the interaction between aging and sex hormones, which exert well-documented but mechanically uncharacterized effects on brain structure and extracellular matrix composition (McEwen et al., 2001; Hao et al., 2006; Brinton, 2009). In a recent study, we showed that hippocampal damping ratio increased between proestrus and estrus phases of the adult female rat estrous cycle (Milbocker et al., 2026). These dynamics mirror known changes to cellular architecture and hormone receptor expression that occur during this characteristic steep drop in estrogen production (Woolley et al., 1990; Klintsova et al., 1995). This finding highlights the sensitivity of brain MRE to nuanced changes to brain health in women, but also to cellular changes occurring across short times. More studies like these on normal, healthy animals are needed to define associations between MRE and histology, but they also need to be paired with behavior, which to date has not been explored concomitantly with MRE (Figure 2).

**Figure 2:**
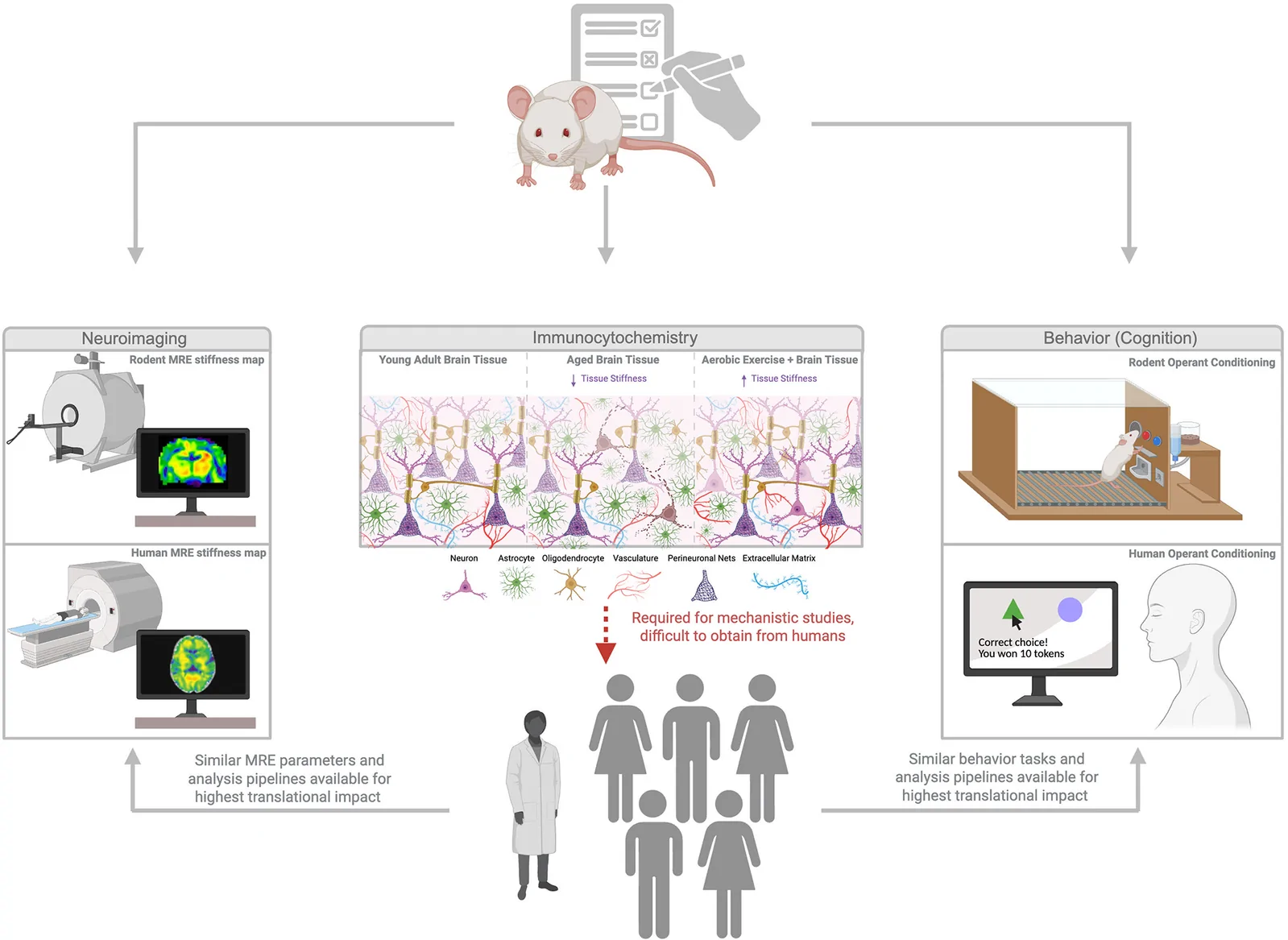
A translational framework for preclinical rodent brain MRE. Brain mechanics measured by MRE **(left)** can be linked to their cellular and matrix basis by immunocytochemistry of the same tissue **(center)** and to cognition by behavioral testing **(right)**. The central histological analysis provides mechanistic evidence difficult to obtain in humans and allows for interpreting results, while shared MRE parameters and behavioral tasks (arrows) support translational impact. “Translational framework for preclinical rodent brain MRE” by K.A. Milbocker created in BioRender (https://shorturl.at/3P352) is licensed under CC BY 4.0.

## 5 Gaps and opportunities to study mechanical structure-function relationships using preclinical MRE

Rodent MRE has so far shown that brain mechanics change with pathology and how, but has provided limited insight into healthy brain structure and function. Rodents allow what human studies cannot through pairing *in vivo* imaging with post-mortem analysis of the same tissue, and the ability to measure the response to experimental manipulation. The sections below outline how to employ preclinical rodent MRE to probe mechanics to identifying the cellular basis of the mechanical signal, linking it to behavior, and resolving the timescales over which it shifts.

### 5.1 Identifying the microstructural basis of MRE changes in the normal brain

The most fundamental gap is the absence of a quantitative correspondence between MRE parameters and the cellular and matrix composition of normal brain tissue. A more complete approach would pair *in vivo* MRE acquisitions with multimodal post-mortem analysis of the same tissue employing quantitative immunohistochemistry for cell-type-specific markers, electron microscopy for myelin sheath thickness and ultrastructural density, and matrix-specific stains. Studies on rodent models, however, are not expected to result in sufficient variability to identify the same structure-function relationships from cross-sectional analyses as in the human brain. Longitudinal observational studies across aging or development also likely encompass too many concurrent changes in cells, mechanics, and function to determine their interactions with specificity, as illustrated by the results from Guo et al. described above (Guo et al., 2019). However, rodent models offer opportunity for controlled interventions that can be applied in relatively short time frames compared to humans (Milbocker et al., 2024a).

Exercise and environmental enrichment are examples of classic intervention paradigms to modify brain structure and function in rodents. The cellular consequences of these interventions are well-established, including neurogenesis (Kempermann et al., 1997; Van Praag et al., 1999), angiogenesis (Cotman, 2002; Swain et al., 2003), dendritic spine complexity (Eadie et al., 2005; Redila and Christie, 2006), extracellular matrix remodeling (Foscarin et al., 2011; Smith et al., 2015), and myelination (Bloom et al., 2022; Milbocker et al., 2023), each of which is a plausible mechanical contributor or suggested by previous preclinical MRE studies. An applied intervention results in the variance that homogeneous, cross-sectional cohorts lack, and longitudinal measurements compared to baseline allow for within-animal statistics that are further enhanced by control conditions to remove confounders. Further, graded exercise intensity or enrichment complexity, or the time of intervention, permits dose-response testing. Where a natural aging trajectory affects cells, matrix, and vasculature together, a controlled intervention affects only a subset, and if MRE measurements in the same animals are also affected, narrows the candidate contributors of each mechanical parameter.

However, such interventions are still not uniquely selective in affecting brain microstructure, and confirming such effects may require selective perturbation. Conditional knockouts of structural proteins, viral-vector-driven cell ablation, and pharmacological matrix disruptions each permit manipulation of a single candidate mechanical driver while holding others approximately constant. Demyelination (with cuprizone (Zhan et al., 2020) or lysolecithin (Dehghan et al., 2021)), neuroimmune upregulation (lipopolysaccharide (Qin et al., 2007), polyinosinic:polycytidylic acid (Altahrawi et al., 2026), or experimental autoimmune encephalomyelitis (Constantinescu et al., 2011)) or extracellular matrix modulation (chondroitinase ABC for perineuronal net digestion (Gogolla et al., 2009), hyaluronidase, MMP-9 modulators (Lorenzo Bozzelli et al., 2018)) may be applied and compared with MRE measurements to confirm the effect of any identified or hypothesized mechanical contributor. We do note that care must be taken in administering such manipulations where the level of disruption is physiologically relevant to the healthy brain, and not an advanced disease stage, as the tissue microstructure-to-mechanics relationship is likely nonlinear (Urbanski et al., 2019).

### 5.2 Entry points for studying brain tissue mechanics and function in rodents

Investigating the mechanical structure-function relationship observed in humans but with rodent models is a logical approach, but identifying appropriate behavioral paradigms for rodents is challenging. Human MRE-cognition studies typically pair a single scan with a testing session encompassing relatively short behavioral tasks and treat performance as a stable characteristic of the participant. Rodent assays, on the other hand, generally require days to weeks of training before performance can be tested, with such an interval corresponding to a meaningful amount of animal aging or development, and that training is itself an experience that remodels the tissue under measurement. Task correspondence is a further challenge as rodent and human assays generally differ in task structure to engage specific brain regions or networks, and many human measures have no established rodent analog. The cross-sectional snapshot therefore cannot simply be reproduced in a smaller animal.

One well-defined behavioral paradigm for rodents offers an entry point for studies comparable to those in humans that requires shorter pre-training. Fear and operant conditioning engage focal hippocampal-amygdalar-pre-frontal circuits with well-characterized potentiation and remodeling dynamics that can be predicted and examined with both MRE and cellular analysis (Curzon et al., 2009). Importantly, fear acquisition occurs within a single session, making it one of the few paradigms whose learning phase and testing phases can be reasonably considered as a single time point that can be compared with a corresponding MRE scan (Maren, 2008). While this is a single assessment, nonetheless the relationships between cells, mechanics, and function would yield critical information to interpret the structure-function relationships broadly. More expansive cognitive batteries with tasks spanning hippocampal-dependent (Morris water maze, novel object recognition) and pre-frontal-dependent (rule learning, attentional set-shifting) functioning would test regional specificity and generalizability of observed relationships, but with experimental designs employing proper control groups to account for the large learning and testing interval.

These paradigms would also help answer the specific question of why some MRE-cognition relationships emerge in stiffness and others in damping ratio, which is likely a reflection of the cellular features driving each parameter. Stiffness is often referred to as reflecting tissue composition while damping ratio reflects tissue organization (Hiscox et al., 2021; McIlvain, 2024), which is a simple interpretation based on foundational biophysical experiments and modeling by Sack et al. (Sack et al., 2013). Other physics-based interpretations of the MRE-measured damping ratio may point to a similar idea (Annio et al., 2024). In this case, stiffness may be derived from the number of each component and their stiffness, while damping ratio may better capture aspects of glial cells, vasculature, and extracellular matrix that give tissue its organized structure—but this is largely conjectural. Such rodent MRE paradigms offer an opportunity to explore and define how and when these two mechanical parameters may be predictive of cognitive function in humans.

### 5.3 Dynamic vs. static mechanics

Rodent models also allow the possibility to explore deeper questions about the relationships between mechanics and cognition, such as whether MRE-detectable mechanics are primarily slow structural properties or can shift on the timescale of cognitive engagement itself. The cellular events of learning provide a natural test as they span the range of spatial and temporal scales needed to dissect this distinction. For instance, repeated synaptic activation during long-term potentiation drives dendritic spine growth and maturation (Harris et al., 2003). Activity-dependent release of matrix metalloproteinase-9 (MMP-9) at potentiated synapses cleaves perisynaptic extracellular matrix and is required for late-phase hippocampal long-term potentiation and contextual fear memory consolidation (Meighan et al., 2006; Nagy et al., 2006). Perineuronal net remodeling reopens windows of plasticity and has been exploited to erase fear and drug associations (Gogolla et al., 2009; Xue et al., 2014). Learning-dependent oligodendrogenesis and myelin sheath remodeling alter axonal conduction and tissue microstructure along behaviorally activated circuits (McKenzie et al., 2014; Bacmeister et al., 2022). Astrocytic perisynaptic processes retract from potentiated synapses during memory consolidation, permitting synapse enlargement and reshaping local neuropil architecture (Ostroff et al., 2014). Complement-mediated microglial pruning selectively eliminates synapses and modulates memory strength (Stevens et al., 2007; Wang et al., 2020a; Schafer et al., 2025). These processes operate on timescales ranging from minutes (MMP-9 release, astrocytic process retraction) to days and weeks (myelination, angiogenesis, perineuronal net remodeling), and each is a plausible contributor to the mechanical properties of tissue.

There is also the opportunity to consider these relationships in terms of active mechanosensing beyond the interpretation of mechanics solely as a passive reflection of the tissue structure. Astrocytic Piezo1-mediated mechanosensing supports neurogenesis and spatial memory in the hippocampus (Chi et al., 2022; Zheng et al., 2023), making tissue mechanics a potential driver of associated functions. As the field of brain mechanobiology and mechanosensing expands (Tyler, 2012; Hall et al., 2021), MRE provides a technique to study *in vivo* brain mechanics and preclinical rodent models are ideal for linking these observations across scales.

One emerging area of note is the nascent functional MRE methodology (fMRE) (Patz et al., 2019; Lan et al., 2020; Palnitkar et al., 2024). fMRE extends this question to the shortest timescales by probing whether the measured MRE mechanical properties shift within seconds of blood oxygenation levels (an analog output for neuronal activity), and possibly faster with effects driven by changes in cell mechanics rather than vascular effects. Such rapid mechanical fluctuations would implicate immediate and reversible changes in vascular and cellular compliance rather than structural remodeling. The approach remains technically demanding and at the proof-of-concept stage, but opens a distinct mechanistic window that would bracket the full temporal range over which brain mechanics and cognition may interact.

## 6 Conclusions

In this work, we provide the background and motivation for preclinical rodent brain MRE studies that can resolve the cellular basis of *in vivo* brain mechanics, and of the mechanical signatures that track cognitive function. The preclinical brain MRE field has made considerable advances in mechanics and disease, but a focus on models of the healthy brain and normal behavior would sharpen what is known about non-pathological change. The ability to observe mechanical change over time, occurring naturally or in response to intervention, further offers access to tissue dynamics not easily studied in humans, and with it new translational applications of MRE. Ultimately, such lines of research would enhance the sensitivity of MRE with interpretability of the resulting brain mechanics measures.

## Data Availability

The original contributions presented in the study are included in the article/supplementary material, further inquiries can be directed to the corresponding author.
